# Antimicrobial, Optical and Mechanical Properties of Chitosan–Starch Films with Natural Extracts

**DOI:** 10.3390/ijms18050997

**Published:** 2017-05-05

**Authors:** Jessica I. Lozano-Navarro, Nancy P. Díaz-Zavala, Carlos Velasco-Santos, Ana L. Martínez-Hernández, Beatriz I. Tijerina-Ramos, Margarita García-Hernández, José L. Rivera-Armenta, Ulises Páramo-García, Adriana I. Reyes-de la Torre

**Affiliations:** 1 Tecnológico Nacional de México-Instituto Tecnológico de Ciudad Madero, Centro de Investigación en Petroquímica, Prolongación Bahía de Aldair, Ave. de las Bahías, Parque de la Pequeña y Mediana Industria, Altamira, Tamaulipas C.P. 89600, Mexico; npatdiaz@hotmail.com (N.P.D.-Z.); betijerina@hotmail.com (B.I.T.-R.); margarita.garcia@itcm.edu.mx (M.G.-H.); jlriveraarmenta@itcm.edu.mx (J.L.R.-A.); uparamo@itcm.edu.mx (U.P.-G.); adriana.reyes@itcm.edu.mx (A.I.R.-d.l.T.); 2 Tecnológico Nacional de México-Instituto Tecnológico de Querétaro, División de Estudios de Posgrado e Investigación, Av. Tecnológico s/n esquina Gral. Mariano Escobedo, Col. Centro Histórico, Querétaro, Querétaro C.P. 76000, Mexico; cylaura@gmail.com (C.V.-S.); almh72@gmail.com (A.L.M.-H.)

**Keywords:** chitosan–starch, natural extracts, antimicrobial activity, optical and mechanical properties

## Abstract

Natural extracts possess several kinds of antioxidants (anthocyanins, betalains, thymol, carvacrol, and resveratrol) that have also demonstrated antimicrobial properties. In order to study these properties, extracts from cranberry, blueberry, beetroot, pomegranate, oregano, pitaya, and resveratrol (from grapes) were obtained. Growth inhibition tests of mesophilic aerobes, coliforms, and fungi were conducted in films prepared from the extracts in accordance with Mexican Official Norms (NOM). Optical properties such as transparency and opacity, mechanical properties, and pH were also analyzed in these materials. The films with beetroot, cranberry, and blueberry extracts demonstrated the best antimicrobial activity against various bacteria and fungi in comparison with unmodified chitosan–starch film. This study shows that the addition of antioxidants improved the antimicrobial performance of these films. It was also found that antimicrobial properties are inherent to the films. These polymers combined with the extracts effectively inhibit or reduce microorganism growth from human and environmental contact; therefore, previous sterilization could be unnecessary in comparison with traditional plastics. The presence of extracts decreased transmittance percentages at 280 and 400 nm, as well as the transparency values, while increasing their opacity values, providing better UV–VIS light barrier properties. Despite diminished glass transition temperatures (*T*g), the values obtained are still adequate for food packaging applications.

## 1. Introduction

In recent decades, human health has been increasingly threatened by microbiological contamination; this can be seen in the spread of food-borne infections and communicable diseases that has occurred recently. Therefore, it is necessary to find, develop, and improve antimicrobial natural materials capable of inhibiting the proliferation of various microorganisms. Several research studies have shown that chitosan and other several natural antioxidants are natural biocides that are promising candidates for solving this problem [1].

Chitosan is obtained by the *N*-deacetylation of chitin, which is the second most abundant natural polysaccharide in nature. Due to its good biocompatibility, it is widely applied in the pharmaceutical, agricultural, food, nutritional, and biomedical industries, in addition to other applications such as sewage treatment. In recent years, the bactericidal and fungicidal features of chitosan have drawn wide attention [2]. Chitosan is not soluble in pure water or organic solvents, but it is soluble in aqueous solutions of organic or mineral acids. Chitosan-based materials can be used as degradable and edible films or coatings. Starch has been used to produce biodegradable films, partially or totally substituting plastic polymers, because of its low cost and renewability. However, the broad applications of pure starch films are limited by the water solubility and fragility of the material [3].

There are various antioxidant substances found naturally in foods that slow the oxidation reactions in cells from which harmful free radicals originate. Therefore, antioxidants play a key role in reducing cardiovascular disease, tumors, and neurodegenerative diseases. Furthermore, they strengthen the immune system. These substances have been studied in recent years because of their biocidal and antiseptic properties, as well as their abundance in nature [4]. Natural antioxidants such as anthocyanins, betalains, thymol, carvacrol, and resveratrol are known for their excellent antimicrobial properties. Anthocyanins are water-soluble pigments with glycoside features that are widely distributed in the plant kingdom. They are responsible for the reddish, orange, bluish, and purplish coloration of grapes, apples, roses, strawberries, and many plant products, mainly fruits and flowers [5]. Betalains are natural water-soluble pigments with nitrogen in their structure; they are synthesized from the amino acid tyrosine. Betalains are divided into two groups: betacyanins, which produce a red color, and betaxanthins, which provide a yellow color [6]. Thymol is a colorless, crystalline substance with a characteristic odor that is present in the essential oils of thyme or oregano. Thymol belongs to the group of terpenes and it has disinfectant and fungicidal features. Carvacrol is an isomer of thymol [7]. Resveratrol is an antioxidant produced by more than 70 species of plants in response to stressful situations (ultraviolet radiation, fungal infections, etc.). It is present in many foods, such as grapes [8].

Mesophilic aerobic bacteria grow in the presence of air and temperature ranges between 20 and 45 °C; their investigation in food science is very important, because they are indicators of food contamination due to contaminated raw materials, and inadequate cleaning and disinfection processes. Also, their presence indicates the existence of conditions that can favor the growth of pathogens such as *Clostridium botulinium*, *Cl. Perfringers*, *Bacillus mesentericus*, *B. subtilis*, *Lactobacillus lycopersici*, *L. pentaceticus*, etc. High counts of these bacteria indicate that food will soon spoil because of these microorganisms [9].

The presence of total coliforms is an indicator of potential fecal human contamination and the presence of enteric pathogens [10]. *Penicillum notatum* is a fungus that grows quickly, between 3 and 7 days. It rarely causes fungal disease in compromised patients [11]. *Aspergillus fumigatus* can cause allergic aspergillosis, which causes granulomas in the lungs. Rarely does the organism cause diseases in humans [12]. *Aspergillus niger* has various industrial applications. It can rarely cause external otitis and allergic aspergillosis [13].

The innovative purpose of this study is to intensify the biocidal properties of chitosan–starch films against food-contaminant-indicator microorganisms (mentioned here) by using organic and nontoxic materials, such as natural extracts, with demonstrated antimicrobial properties that are compatible with the matrix film. By using simple and cheaply obtained extracts from renewable resources, this innovation may also reduce costs. These new biomaterials are promising candidates for food packaging because of their lower transparency, higher opacity, flexibility, and adequate operating temperature.

## 2. Results and Discussion

### 2.1. Antimicrobial Activity

The antimicrobial activity of each film was observed using two series of three tests. A film was considered biocidal if it prevented the growth of microorganisms not only in its surface but also in its periphery. The antimicrobial activity of these films cannot be explained perfectly, but some researchers have proposed several mechanisms. The antimicrobial activity of chitosan–starch with natural antioxidants from natural extracts may be attributable to the following mechanisms. 

The electrostatic interaction between positively charged chitosan and some bacteria with negatively charged cellular membranes (such as certain types of aerobic mesophilic and coliform bacteria) significantly alters the barrier properties of these membranes. This implies the modification of nutrients and waste flow, resulting in the bacterium’s elimination. The phospholipids present in the cellular membranes of Gram-negative bacteria interact with the NH- groups of the chitosan, causing the bacteria to lose cellular material. The chelating capacity of the chitosan can affect microbial growth. The use of acetic acid as a chitosan solvent can improve the antimicrobial activity of chitosan according to a study that showed that organic acids with low carbon numbers produce better antibacterial solutions [14]. 

The external membranes of bacteria protect them against polar organic compounds, which are harmful to them [15]. Consequently, it is possible that cell membranes are broken by the action of chitosan, promoting the entry of natural antioxidants into the cells. The antioxidants have antimicrobial properties and can therefore increase the antibacterial properties of chitosan. The interaction between positively charged amino groups of chitosan (positive charge is caused by the acid solvent) and negative residues in the fungi walls changes the permeability of the fungal plasmatic membrane, altering its main functions. The presence of chitosan inhibits some enzymatic synthesis on fungi and can favor the occurrence of cytological alterations [16].

Carvacrol is capable of breaking the external membrane of Gram-negative bacteria, causing the exit of lipopolysaccharides and increasing the permeability of the cytoplasmic membrane [17]. The presence of carvacrol stimulates the removal of adenosine triphosphate (ATP) from the cell, inhibiting enzymes and decreasing the proton motive force [7,18]. 

Thymol is capable of disintegrating the external membrane of Gram-negative bacteria, resulting in the elimination of lipopolysaccharides and increased cytoplasmic membrane permeability. Thymol changes the membrane permeability and permits the loss of essential compounds such as ions, ATP, nucleic acids, and amino acids [7]. In our tests, most of the films with extracts demonstrated effectiveness against the presence of microorganisms, which is considered the main cause of food decomposition.

#### 2.1.1. Previous Contamination Test

After incubation of the microorganisms for a proper period, the number of colony-forming units (CFU) developed in each case was counted. The limits specified in the relevant standards were considered, and we found that very few films presented numerous colonies. The results and film nomenclature are presented in Table 1. The CFU numbers presented in Table 1 were counted visually. Films were considered contaminated if they presented a CFU number equal to, or higher than, the number considered in the section 10 of each applicable norm. For mesophilic bacteria, plates with >25 CFU were considered contaminated [19]. For coliform bacteria, plates containing ≥1 CFU were considered contaminated [20]. For fungi, plates with >10 CFU were considered contaminated [21]. In the case of mesophilic aerobic bacteria, we found that most of the films (with the exception of QSG2, QSG5, and QSO5 in one of two tests) inhibited or reduced the growth of mesophilic aerobic bacteria. In the case of coliforms, it was observed that most of the films (with the exception of QSAm0.5, QSB0.5, and QSP0.5 in one test) are sterile to coliforms, revealing that even if they are in contact with this kind of bacteria, they do not become contaminated. In the case of fungi, it was observed that most of the films presented better inhibition of environmental fungi than the control chitosan–starch film (QS2) (with the exception of QSA5, QSB0.5, QSO5, and QSP5). In summary, the presence of natural extracts provided the films with a better resistance to environmental microorganisms and human contact, which implies that most of them, unlike traditional biocide polymers, will not require a previous sterilization process. 

#### 2.1.2. Activity against Aerobic Mesophilic Bacteria 

Most foodborne pathogenic bacteria are mesophilic (they grow in a range of temperature similar to that of the human body). For this reason, it is important to prove the inhibition capacity of our films against aerobic mesophilic bacteria (they are indicators of food contamination, but they are not pathogens in a strict sense). The control chitosan–starch film did not show any inhibitory effect on aerobic mesophilic bacteria. However, it was observed that all films with cranberry, blueberry and beetroot extracts, QSG2, QSG5, QSO2, QSO5, QSP0.5, and QSR5, showed an inhibition effect on these microorganisms in most cases. These results mean that the addition of antioxidants significantly improved the antibacterial activity of the films, especially in the case of anthocyanins, since they are more stable than any other kind of antioxidants. The antibacterial activities of chitosan–starch films with natural antioxidants after 48 h is shown in Table 2 (in accordance with section number 11—Test report of norm NOM-092-SSA1-1994 [19]—we had to report the bacterial growth at that time). We observed a significant improvement in antibacterial activity due to the addition of natural extracts, and the same behavior was observed in the research of Duran et al., which achieved improved inhibition of aerobic mesophilic bacteria by using natamycin, nisin, grape seed extract, and pomegranate extract [22]. A similar result was obtained by Yuan et al., who reduced the presence of aerobic bacteria in stored shrimp by the addition of pomegranate peel extract to a chitosan solution [23]. Paparella et al. improved antilisterial activity by using a mixture of chitosan and oregano essential oil [24]. Section 2.1 describes the antibacterial mechanisms. 

#### 2.1.3. Activity against Coliforms

Coliforms are Gram-negative bacteria that are indicators of possible food and water contamination; the main bacterium of this group is *Escherichia coli.* The control chitosan–starch film showed little inhibitory effect on coliform bacteria (only in one test). However, it was observed that all films with cranberry and blueberry, QSG5, QSR0.5, and QSR5, showed the inhibition of these microorganisms in most cases. The other films showed medium antibacterial activity. The beetroot films showed the same results as QS2. These results reveal that the addition of anthocyanins and resveratrol significantly improved the inhibition effect on this group of bacteria. Anthocyanins were more effective than betalains and thymol/carvacrol, because they are more stable to changes. The antibacterial activity of chitosan–starch films with natural antioxidants after 24 h is shown in Table 2 (in accordance with section 11—Test report of norm NOM-113-SSA1-1994 [20]—we had to report the bacteria growth at that time). In accordance with the research of Shen et al. and Pranoto et al., we observed that chitosan in combination with natural extracts can inhibit the growth of *E. coli* (the representative bacteria of coliforms) [25,26]. See Section 2.1 for an explanation of the antibacterial mechanisms.

#### 2.1.4. Activity against Fungi 

Fungi are a huge group of microorganisms widely distributed in the environment. In this study, we observed inhibition activity against three kinds of fungi: *Penicillum notatum*, *Aspergillus niger*, and *Aspergillus fumigatus*. The *Penicillum* fungi and the *Aspergillus* fungi exist on most surfaces; they grow during food decomposition (for example in bread and fruits). They are not potentially dangerous to humans (except in those with weak immune systems). The control chitosan–starch film showed little inhibitory effect on coliform bacteria (only in two tests). However, it was observed that all the beetroot films, QSA5, QSAm2, QSAm5, QSG5, QSO5, and QSP0.5, showed the inhibition of these microorganisms in most cases. The resveratrol films showed medium antibacterial activity. The other two pomegranate films showed low antifungal activity. These results demonstrate that the addition of anthocyanins from cranberry, blueberry, and pomegranate, betalains from beetroot, and thymol/carvacrol from oregano in major quantities, significantly improved the inhibition of these groups of fungi. The antifungal activity of chitosan–starch films with natural antioxidants after five days (120 h) is shown in Table 2 (in accordance with section number 11—Test report of norm NOM-111-SSA1-1994 [21]—we had to report the fungi growth at that time). The control chitosan–starch film showed an inhibitory effect on fungi. In the research of Martínez-Camacho et al., Plascencia-Jatomea et al., and Ture et al., it was observed that chitosan does not totally inhibit fungal growth, although having a medium molecular weight, chitosan is a good fungistatic agent [27,28,29]. See Section 2.1 to read about the fungistatic mechanisms.

### 2.2. Thickness Measurement

The films had a homogeneous appearance, and they were easily peeled off from a polystyrene tray (except for QSP5, which was sticky). The samples presented different colorations due to the presence of different extracts. We observed similar behavior to that noticed by Benavides et al., which indicated that the addition of oregano essential oil produced bactericidal alginate films that were significantly thicker (0.031–0.038 mm) [30]. We used higher extract concentrations to prove that aqueous extracts can increase the film thickness. According to Qin et al. [31], the thickness of chitosan/montmorillonite films increased with the addition of pomegranate rind extract, because of its high molecular weight polyphenolic compounds. The films were prepared using the same amount of chitosan solution; the differences between the films were the quantities of montmorillonite and pomegranate rind extract. Nevertheless, the authors indicated that the increment of the film thickness is directly related only to the increment of the pomegranate extract [31]. Our samples showed a similar behavior to that described by Espitia et al. in their investigation, where the presence of apple skin polyphenols and thyme essential oil compounds increased the thickness of the control film [32]. In our investigation, the films were prepared and stored in a room with controlled temperature, and the quantities of each compound of the films were carefully measured in order to reduce the influence of the preparation and storage conditions on the thickness increase. In our research, the thickness increase can be attributed to the presence of natural antioxidants and to the variation of the water content in the starch solution.

Table 3 indicates the average film thickness of each sample. We observed that the addition of extracts enhanced the average thickness of the film (except QSB0.5). In some cases, the higher the extract concentrations, the thicker the film (especially for cranberry, blueberry, and beetroot). The thickness increase could be caused by the presence of compounds in the extract such as polysaccharides, carboxylic acids, antioxidants, etc., creating a more complex matrix. Thickness was confirmed by optical properties improvement and storage modulus decreases (both facts are related to film thickness). 

### 2.3. Optical Properties (Transparency and Opacity)

To determine the light transmission properties of each film, we considered the percentage of transmittance at 280 nm, which corresponds to the transmission of UV light (this kind of light causes lipid oxidation in food). It was found that the addition of extracts gave the chitosan–starch films better barrier properties against ultraviolet light (except for QSR5). At 400 nm, the films with extracts presented lower transmittance percentages, indicating better light barrier properties in comparison with QS2 (except for QSR2). These results are presented in Table 4. 

Table 4 shows the average film transparency of each sample. All the films (except for QSB0.5) presented lower transparency than QS2 (the control film). In most cases, the higher the extract content, the lower the film transparency.

At higher transparency, the film must have lower opacity. In Table 4, the average film opacity of each sample is reported. It is observed that the addition of extracts increased the opacity in comparison with QS2 (the control film), which means that the extracts improved the light barrier properties of the films (except in the case of QSO2). These results indicate that these films are effective against the second cause of food decomposition: lipid degradation. We observed different behavior than that of the films studied by Kanatt et al. [33]; we used higher quantities of extract and we obtained thicker films. This means that the quantity of extract has a significant effect on film thickness and, consequently, on its optical properties. Additionally, the film color had a significant effect on the optical properties. We observed that the control film QS2 is more crystalline and less thick than the films with extracts. For more details about film crystallinity, see Section 2.5.1.

### 2.4. pH Measurement

In Table 5, the pH values obtained for each film compound are reported. These results correspond to the average values of two tests. The following information was considered: anthocyanins are stable in acid media [5], betalains have their maximum stability at pH 5–6 [6], and resveratrol is stable in acid media [34]. These results confirm that acetic acid is a good solvent for chitosan (which has a low pH value). It is observed that the extracts with lower pH values (cranberry, blueberry, pomegranate, and beetroot) corresponded to films with better antimicrobial activity. 

Table 6 presents the pH values after the synthesis (day one). With lower pH, antimicrobial activity is better. The most effective films during the antimicrobial tests (see Table 2) were those that had a lower pH after the synthesis (except pitaya). This may be related to the fact that antioxidants are more stable in acid media. 

Table 6 also shows the pH values obtained when the films were dry and ready to use (day 15). We observed an increase in pH values, which could be a consequence of the chitosan solvent (acetic acid) evaporation during the drying period. It is possible that the addition of extracts causes a higher pH in comparison to QS2 due to the presence of certain extract compounds such as water.

The pH test values confirmed the presence of antioxidants as follows. At pH values higher than 7, anthocyanins were degraded, depending on their substituent groups. At pH values between 4 and 6 (all the films are in this range) the four structural forms of antioxidants can coexist. For this reason, it is possible to observe certain colorations in the cranberry, blueberry, and pomegranate films. However, there is color degradation in the presence of natural/artificial light [35].

Betalains are relatively stable in the pH range between 3 and 7 (the beetroot and pitaya films have pH values in this range) [36]. The optimum pH range for betalain stability is 5–6 [37]. Again, it was confirmed that films with lower pH values had the best results during the antimicrobial tests (see Table 2). This can be related to the fact that the antioxidants are more stable in acid media, which provides a good interaction with the chitosan–starch blend and potentiates the antimicrobial activity. The acetic acid had no relevant role on antimicrobial activity, because it was not an experimental variable: all the films, including the control film QS2, contained the same quantity of diluted acetic acid, and we observed that QS2 had weaker antimicrobial activity than the films with extracts (see Table 2). The same behavior was observed in the research of Gupta et al., they concluded that the addition of poly (acrylamide)/ZnS improves the antimicrobial activity of chitosan, not the acetic acid presence [38] and Moreira et al., they indicated that the addition of sodium caseinate enhances the antimicrobial activity of chitosan, not the acetic acid [39]. In both researches, chitosan solutions were prepared using acetic acid solution at 1% in all their samples. Also, the pH increment between day 1 and 15 can be attributed to the partial volatilization of solvents such as water and acetic acid. 

**Figure 1:**
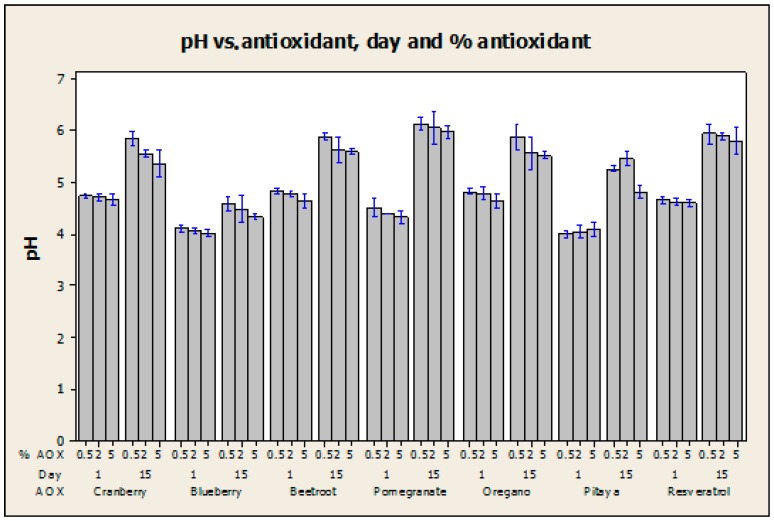
Effect of the concentration extract, type of antioxidant, and measurement day on the pH values of the films. Each data point represents the mean ± standard deviation (SD); Analysis of variance (ANOVA), Tukey test, *p* < 0.05.

Analysis of variance (ANOVA) provided statistical evidence about the effect of the antioxidant, its concentration, and the measurement day on the pH values. If the calculated *p* value of a certain factor is less than the significance level, the null hypothesis of no difference among means, or no interaction among factors, is rejected, as displayed in Table 7. Because the three independent variables proved to produce at least one significant difference in the mean pH, a post hoc test (Tukey’s method using 95% confidence intervals) was employed to explore which levels of the three factors could be considered as the sources of those differences. For instance, the statistical analysis allowed us to discover that the mean pH does not significantly differ among samples that contain resveratrol, pomegranate, beetroot, oregano, and cranberry (means of 5.244, 5.228, 5.220, 5.194, and 5.141, respectively, calculated considering just the antioxidant (AOX) as the cause of variation, according to the Tukey’s method); nevertheless, an important difference in the mean pH values was detected as a result of the pitaya and blueberry incorporation (means of 4.610 and 4.262, respectively). In addition, the test revealed that the mean pH does not change considerably between the concentrations of 0.5% and 2% of antioxidant (means of 5.081 and 4.996, respectively), nor between the concentrations of 2% and 5% (4.996 and 4.880, respectively); however, at the confidence level of the Tukey’s test, a representative change in the mean pH took place between the concentrations of 0.5% and 5% (5.081 and 4.880, respectively). It was also concluded that the day when the pH of the film is measured has an important effect on the mean value (means of 5.496 for day 1 and 4.475 for day 15), which is attributed to the evaporation of solvents during the drying period after the synthesis procedure. The possible combined interaction of the experimental factors was correspondingly studied to demonstrate which effects were dependent on each other. The analysis showed that just two joint interactions (AOX-day and AOX-%·AOX-day) induced significant differences among the mean pH results. Also, the effect of concentration extract, type of antioxidant and measurement day on the pH values of films can be observed in Figure 1.

### 2.5. Dynamic Mechanic Analysis (DMA)

#### 2.5.1. Storage Modulus (E’) 

**Figure 2:**
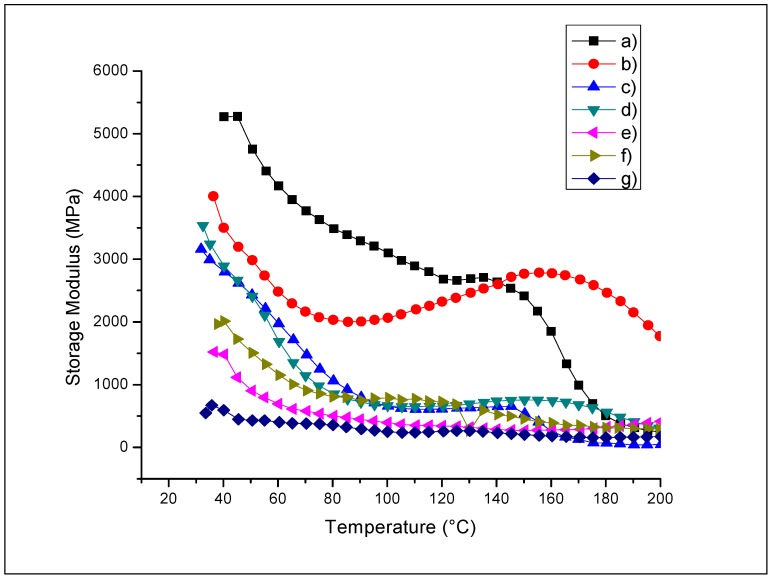
Storage modulus (E’) of the chitosan–starch films: (**a**) QS2; (**b**) QSA5; (**c**) QSAm5; (**d**) QSG5; (**e**) QSO5; (**f**) QSP0.5; (**g**) QSR5.

The results of the storage modulus (E’) are presented in Table 8. In Figure 2, the storage modulus curves of QS2, QSA5, QSG5, QSO5, QSP0.5, and QSR5 are shown. The addition of natural extracts significantly reduced the storage modulus values; this means that films with extracts were more flexible than QS2. It was also observed that when the concentration of extracts increased, the storage modulus decreased. However, the elastic behavior depended on the extract type; thus, not only opacity and antimicrobial activity could be manipulated in these films, but also a different level of rigidity could be achieved depending on the extract used. Manipulating rigidity may benefit possible applications in food coatings because the rigid film is related to brightness behavior.

In the E’ curves of the QS2, QSA0.5, QSA2, QSA5, QSAm5, QSG5, QSP0.5, and QSP5 films we observe three regions, in accordance with the investigation realized by Al-Sagheer et al. (2011) [40]. The first region comprises the lowest temperatures, 80–90 °C (crystalline region), where the molecules’ movements are restricted. The second region is the transition between the crystalline and elastic regions, where the main and lateral chain rotations are realized by increasing the temperature from 80–90 °C to 130–150 °C. In this region, the storage modulus presents an increment and a subsequent decrease (with the exception of QS2, QSAm5, and QG5; in the first two, only the decreasing process occurs and in the third one, only the increasing process) due to the presence of starch, which acts as a plasticizer. The third region is known as the viscoelastic region, and its deformation through time gives the glass transition temperature or *T*g. This region is situated between 130–150 °C and 200 °C. We observed little increment of the storage modulus, which can be attributed to the rapid alignment of the polymeric chains under sinusoidal stress [40]. 

The QSO5 and QSR5 films did not show such behavior. It was observed that a higher extract concentration lowered the storage modulus value. The QSA0.5, QSA2, QSA5, QSG5, and QSP0.5 films had good E’ values and presented better antimicrobial activity than the rest of films analyzed by DMA (Table 2). The plasticizing effect of starch and glycerol is known, but for this work it was decided that they were not experimental variables, since there is a diverse investigation of the effect of plasticizers on chitosan [41]. The quantities of starch and glycerol used in this study were constant. The change in mechanical properties and flexibility of each film can be attributed to the interactions of starch, glycerol, and extracts, which possess antioxidants. These compounds are polyphenols, which contain OH- groups that form various bonds with the polymer matrix (e.g., hydrogen bonds) [33]. According to Bonilla et al., the decrease of storage modulus values in chitosan-based films can be attributed to the incorporation of natural compounds such as antioxidants and terpenes. This decrease is produced by discontinuities in the film structure and the lack of cohesion among polymeric chains. We deduced that our results are considered favorable due to the plasticizing effect of polyphenols from natural extracts, which, as explained by the studies mentioned before, allows a smoother displacement of the polymeric chains during the stretching of the films, and makes possible the undamaged deformations of each film [42]. 

We observed a decrease in the initial storage modulus values (see the E values at 35–40 °C in Figure 2), which is related to a less crystalline material. The decrease in crystallinity benefits the antimicrobial activity (see Table 2). The order of crystallinity is as follows: QS2 > QSA5 > QSG5 > QSAm5 > QSP0.5 > QSO5 > QSR5. A decrement in the film crystallinity reduces the intermolecular forces, giving less rigid and less brittle films. 

#### 2.5.2. Glass Transition Temperature (*T*g)

**Figure 3:**
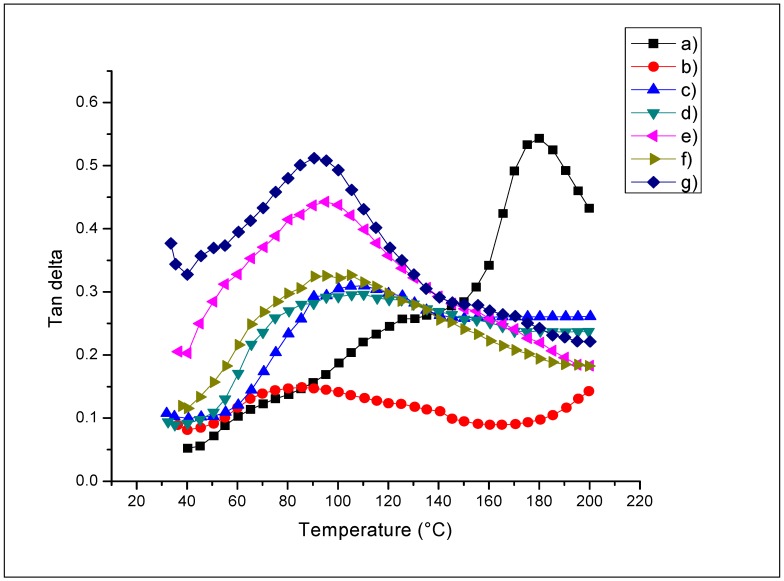
Tan delta (tan δ) of the chitosan–starch films: (**a**) QS2; (**b**) QSA5; (**c**) QSAm5; (**d**) QSG5; (**e**) QSO5; (**f**) QSP0.5; (**g**) QSR5.

The results of tan delta (tan δ) and *T*g are indicated in Table 8. The films analyzed by Dynamic Mechanical Analysis (DMA) showed good antimicrobial activity; see Table 2. Additionally, these films presented an improvement in their optical properties in comparison with QS2; see Table 4. In Figure 3, the tan δ curves of QS2, QSA5, QSG5, QSO5, QSP0.5, and QSR5 are shown. A decrement of the tan δ values is observed in films with natural extracts, indicating a clearly different behavior in the slippage between chitosan and starch chains depending on the natural extract added. The changes in structural behavior of these materials are clearly related to the extracts integrated into the material, and thus, modify the polymer movement depending on the nature of the integrated extract and its concentration. The *T*g of the chitosan solution in acetic acid has been reported at 203 °C [27]. In the chitosan–starch films in this study, a *T*g around 182 °C was found. A decrement of *T*g was observed in every sample due to the different miscibility of each compound. A wider transition zone may indicate a possible reaction between the functional groups (antioxidants) and the film matrix. This reaction may be related to the decomposition of antioxidants, since they are unstable at high temperatures. Thus, as mentioned earlier, the tan δ and *T*g values decrease with the addition of extracts. The *T*g measurement of a polymer blend can give information about the films’ miscibility. A single peak in tan δ curves could be an indication of good miscibility between chitosan–starch and the natural extracts [43]. These materials could be used for food packaging since *T*g, or the operating temperature values, are above 80 °C. 

## 3. Materials and Methods 

### 3.1. Materials and Reagents

For the synthesis of chitosan–starch films, medium molecular weight chitosan with a degree of deacetylation of 85% (commercial compound obtained from shrimp) and rice starch were purchased from Sigma-Aldrich (Toluca, Edo. Mex., Mexico). Glacial acetic acid and glycerol were bought from Fermont (Monterrey, NL, Mexico). Extracts were obtained from raw fruits such as blueberry (130 g), beetroot (1 small beetroot weighing 220 g), and pitaya (1 regular pitaya weighing 277 g). These extracts were obtained from raw fruit pulp at 25 °C and were filtered prior to their addition in the chitosan–starch–glycerol blend. The aqueous extract of oregano was prepared at 5% (*w/v*) using dry oregano and water as a solvent; the solution was prepared at 65 °C for 10 min, with magnetic agitation and further filtration. Commercial organic cranberry and pomegranate juices were used; the extract was filtered at 25 °C before its addition to the blend. Resveratrol capsules were purchased from General Nutrition Centers (Pittsburgh, PA, USA). The resveratrol solution at 5% (*w/v*) was prepared using water as a solvent at 25 °C, after which the solution was filtered. It was very important to obtain the extracts of cranberry, blueberry, beetroot, pomegranate and pitaya at 25 °C in order to preserve their antioxidants [5,6,7,8]. Extracts were prepared in these forms with the purpose of reducing expenses, and avoiding toxic solvents. These aqueous extracts were compatible with the film matrix. For the microbiological methods, standard method agar, potato dextrose agar, and brilliant violet bile agar were purchased from Bioxon (Mexico City, Mexico). Tartaric acid was purchased from Mallinckrodt Chemical (St. Louis, MO, USA).

### 3.2. Synthesis of Films 

The chitosan solution (2%, *w/v*) was prepared in glacial acetic acid solution (1%, *v/v*). The resulting solution was stirred for 24 h at room temperature. The rice starch solution (2% *w/v*) was prepared by heating at 90 ± 2 °C for 20 min with constant stirring. The solution was cooled to approximately 25 °C. For preparing each film, 40 mL of chitosan solution was mixed with 40 mL of rice starch solution. Afterwards, 0.2 mL of glycerol was added as a plasticizer. Each blend was stirred for 5–10 min, and then, poured in a polystyrene tray. The films were dried at 25 °C for 15 days [44]. For the films with natural antioxidants, three fixed quantities (0.4, 1.6, and 4 mL) of each extract were added (corresponding to the 0.5%, 2%, and 5% weights of the total blend). The only caution needed during the synthesis was related to the manipulation of glacial acetic acid, glycerol, and the hot starch solution (90 ± 2 °C). Twenty-two films were prepared: one control film (QS2) and twenty-one films with natural extracts (three films for each extract). The explanation of the code used for each film is given in Table 1. 

### 3.3. Antimicrobial Activity

The agar plate method was used to evaluate the antimicrobial activities of the chitosan–starch films with natural extracts against the control chitosan–starch film. The antimicrobial activities of the films were tested against *Penicillium notatum*, *Aspergillus niger*, *Aspergillus fumigatus*, coliforms, and aerobic mesophilic bacteria, and a previous contamination test was carried out before the addition of these microorganisms in order to ensure that they had no microorganisms prior to the antimicrobial test. For the antimicrobial tests (see Section 3.3.2, Section 3.3.3 and Section 3.3.4), it was necessary to use dilutions to reduce the microbial load for facilitating the microbial counting. It is necessary to be cautious during the preparation of these agars because they are very hygroscopic. Thus, latex gloves and surgical masks were used during all antimicrobial tests. During the antimicrobial tests, the inhibition zone of each film was measured using a Vernier with a millimeter scale, considering the zone of inhibition as the difference between the diameter of the inhibition zone and the diameter of the disk [45]. The measurement was made for each test, and the average values are indicated in Table 2. 

#### 3.3.1. Previous Contamination Test 

This test was carried out to ensure that the films were capable of inhibiting environmental microorganisms prior to the antimicrobial activity tests. This means that the films are capable of inhibiting microorganisms introduced by human manipulation or surface contact. In 5 mL of sterilized distilled water, a 1 cm^2^ film sample was placed for 24 h. Subsequently, 1 mL of this water was added to a petri dish without dilution. Later, the correspondent agar was added. The procedures for growing microorganisms indicated in Section 3.3.2, Section 3.3.3 and Section 3.3.4 were used in this test. 

#### 3.3.2. Activity against Aerobic Mesophilic Bacteria

Standard method agar was used for the growth of aerobic mesophilic bacteria. To prepare 100 mL of agar solution, 2.35 g of agar was dissolved in distilled water. The solution was heated and boiled for one minute under constant stirring. The agar was sterilized in an autoclave at a pressure of 15 pounds for 15 min. Potable water was used as a sample. One ml of sample was added to each petri dish. Next, agar was poured into the petri dishes. Each petri dish was moved seven times to the right, seven times to the left, and seven times up and down to mix the agar and sample. Once the agar solidified, six disks from the same sample/film were placed on it, considering the distance among them. The petri dishes were incubated at 37 °C. The antibacterial activity was observed for two days at 37 °C [19].

#### 3.3.3. Activity against Coliforms 

Brilliant violet bile agar was used for growing total coliforms. To prepare 100 mL of agar solution, 4.15 g of agar was dissolved in distilled water. The solution was heated and boiled for one minute under constant stirring. Agar and dilution tubes (with 10 mL of distilled water) were sterilized in an autoclave at a pressure of 15 pounds for 15 min. Horchata (La Vitrolera, Tampico, TAMPS, Mexico) was used as the sample because this beverage presented a high number of total coliform CFU in previous analyses. One ml of sample was placed in the dilution tube with 10 mL of sterilized water. Subsequently, a second dilution was made using 1 mL of the first dilution in 10 mL of sterilized water. Later, 0.1 mL of the second dilution was added to each petri dish. Next, agar was poured into the petri dishes. Each petri dish was moved seven times to the right, seven times to the left, and seven times up and down to mix the agar and sample. Once the agar solidified, six disks from the same sample/film were placed over it, considering the distance among them. The petri dishes were incubated at 37 °C. The antibacterial activity was observed for one day at 37 °C [20].

#### 3.3.4. Activity against Fungi 

Potato dextrose agar was used for growing fungi. To prepare 100 mL of agar solution, 3.9 g of agar was dissolved in distilled water. The solution was heated and boiled for one minute under constant stirring. Tartaric acid solution at 10% (*w/v*) was prepared. Agar, tartaric acid, and dilution tubes (with 10 mL of distilled water) were sterilized in an autoclave at a pressure of 15 pounds for 15 min. A measurement of 1.4 mL of tartaric acid was added for each 100 mL of agar in order to obtain a pH of approximately 3.5. *Penicillum notatum*, *Aspergillus niger*, and *Aspergillus fumigatus* colonies were used as samples. The fungi colonies were scraped using a sterilized inoculating loop and the sample was placed in a dilution tube. Subsequently, a second dilution was made using 1 mL of the first dilution in 10 mL of sterilized water. One mL of the second dilution was added to each petri dish. Next, agar was poured into the petri dishes. Each petri dish was moved seven times to the right, seven times to the left, and seven times up and down to mix the agar and sample. Once the agar solidified, six disks from the same sample/film were placed on its surface considering the distance among them. The petri dishes were incubated at 25 °C. Antifungal activity was observed for five days at 25 °C in accordance with the norm [21].

The disk diffusion method (as required by the World Health Organization) was used during all antimicrobial tests to study the effectiveness of the films against various microorganisms. A circular sample of each polymer film was placed on agar [46]. 

### 3.4. Thickness Measurement 

Film thickness was measured with a manual micrometer OBI 264105 (range of measurement 0–25 mm) (OBI Asia Trade, Hong Kong, China). Five measurements were taken for each film, one at the center and four around the perimeter. The thickness is reported as the average of the five measurements [33].

### 3.5. Optical Properties Using Ultraviolet–Visible Spectroscopy 

A rectangular sample (4.5 × 0.5 cm) of every film was cut and placed in a quartz cell, which was introduced into a Cintra 303 UV-Vis spectrophotometer (GBC Scientific Equipment, Mexico City, Mexico). Air was used as the reference. Each spectrum was obtained at wavelengths from 200–800 nm, and every film was tested three times. The results were reported as an average of the transmittance percentage (%*T*). The transparency was obtained by using the value of % transmittance at 600 nm (%*T*) and Equation (1) [33].

*T* = −log %*T*_600_/b (1)

where *%T* is the transmittance percentage and *b* is the average film thickness in mm. 

The opacity (*O*) was obtained using the average absorbance at 500 nm. Each film was tested three times, and the opacity was calculated using Equation 2 [33].

*O* = *A*_500_ × b (2)

where *A*_500_ is the absorbance at 500 nm and *b* is the average film thickness in mm.

### 3.6. pH Measurement

In order to observe the relationship between pH and the antimicrobial activity of the films, the pH was determined using a HI 2212 HANNA Instruments pH meter (HANNA Instruments, Woonsocket, RI, USA). To determine the film pH during the synthesis (day one), the electrode was submerged in the film blend. Two pH measurements were made. The results are reported as an average value. To determine the film pH when dry (day 15), the procedure described in the ASTM D 6739 Standard Test Method for Silica—pH Value (Annex A) was used, which coincides with the procedure stipulated in the Mexican norm NMX-F-317-S-1978 (*Determinación de pH en alimentos).* Distilled water was boiled for 10 min in a covered container. Next, 0.5 g of previously pulverized sample was taken and added to a container with 10 mL of that water and the container was covered. The blend was stirred for five minutes at room temperature. Two pH measurements were made at room temperature, and the results are reported as an average value [47]. 

### 3.7. Dynamic Mechanic Analysis (DMA)

To determine the thermomechanical properties and the influence of the extracts present in the structure of the films, a DMA 800 Perkin Elmer dynamic mechanic analyzer (Perkin Elmer, Waltham, MA, USA) was used at a temperature range from 25–200 °C and a ramp of 10 °C per minute in an atmosphere of N_2_. Two tests were performed and the results are presented as average values.

### 3.8. Statistical Analysis

Statistical analyses were performed using Excel (2013 version, Microsoft, Redmond, WA, USA). Multi-factor analysis of variance (ANOVA) using Minitab (16 version, Pennsylvania State University, State College, PA, USA) was used to determine if the formulation, storage period, or the interaction of these effects had a significant impact on the film solution pH. The statistically significant differences between means were evaluated using the Tukey’s test (*p* < 0.05).

## 4. Conclusions 

Based on our results, we conclude that the addition of extracts, especially at concentrations of 2% and 5% *v/v*, increases antimicrobial activity. No previous sterilization is necessary for most of these materials. The addition of antioxidant-containing extracts provides a film with better UV–VIS light barrier properties, which implies higher values of opacity and lower values of transparency. The presence of extracts improves the mechanical properties of the films. Additionally, we observed a thickness increment derived from the extract presence. These desirable characteristics were more frequent in the films with cranberry, blueberry, and beetroot extracts. Most of the films prepared in this study have a potential application in the food packaging industry because they are non-toxic, biodegradable, and cheap materials that can inhibit food contamination/degradation. These materials have lower transparency, higher opacity, and better mechanical properties than the control film QS2. 

Future research directions include the study of extract effects on the morphological, thermal, and structural properties of the films and their relationship with antimicrobial activity, optical, and mechanical properties. 

## Figures and Tables

**Table 1:** Results of previous contamination tests.

Code Sample	Aerobic Mesophilic Bacteria (CFU)			Coliforms (CFU)			Fungi (CFU)		
	Test 1	Test 2	Average Value	Test 1	Test 2	Average Value	Test 1	Test 2	Average Value
QS2	1	4	2.5 ± 2.12	<1	2	1 ± 1.41	10	3	6.5 ± 4.95
QSA0.5	<1	<1	<1 ± 0	<1	<1	<1 ± 0	<1	<1	<1 ± 0
QSA2	<1	<1	<1 ± 0	<1	<1	<1 ± 0	1	1	1 ± 0
QSA5	1	1	1 ± 0	<1	<1	<1 ± 0	9	14	11.5 ± 3.54
QSAm0.5	<1	1	0.5 ± 0.71	1	<1	0.5 ± 0.71	1	2	1.5 ± 0.71
QSAm2	<1	<1	0 ± 0	<1	<1	<1 ± 0	<1	1	0.5 ± 0.71
QSAm5	<1	<1	0 ± 0	<1	<1	<1 ± 0	<1	1	0.5 ± 0.71
QSB0.5	1	1	1 ± 0	1	<1	0.5 ± 0.71	<1	17	8.5 ± 12.02
QSB2	2	<1	1 ± 1.41	<1	<1	<1 ± 0	<1	0	<1 ± 0
QSB5	3	1	2 ± 1.41	<1	<1	<1 ± 0	1	5	3 ± 2.83
QSG0.5	2	3	2.5 ± 0.71	<1	<1	<1 ± 0	8	3	5.5 ± 3.54
QSG2	<1	26	13 ± 18.38	<1	<1	<1 ± 0	2	2	2 ± 0
QSG5	<1	31	15.5 ± 21.92	<1	<1	<1 ± 0	6	7	6.5 ± 0.71
QSO0.5	<1	1	0.5 ± 0.71	<1	<1	<1 ± 0	3	2	2.5 ± 0.71
QSO2	10	<1	5 ± 7.07	<1	<1	<1 ± 0	3	5	4 ± 1.41
QSO5	3	27	15 ± 16.97	<1	<1	<1 ± 0	4	11	7.5 ± 4.95
QSP0.5	<1	2	1 ± 1.41	<1	2	1 ± 1.41	2	<1	1 ± 1.41
QSP2	<1	1	0.5 ± 0.71	<1	<1	<1 ± 0	3	1	2 ± 1.41
QSP5	1	3	2 ± 1.41	<1	<1	<1 ± 0	10	1	5.5 ± 6.36
QSR0.5	<1	<1	<1 ± 0	<1	<1	<1 ± 0	<1	1	0.5 ± 0.71
QSR2	<1	<1	<1 ± 0	<1	<1	<1 ± 0	1	1	<1 ± 0
QSR5	<1	1	0.5 ± 0.71	<1	<1	<1 ± 0	2	1	1.5 ± 0.71

Q: chitosan; S: starch; A: cranberry; Am: blueberry; B: beetroot; G: pomegranate; O: oregano; P: pitaya/dragon fruit; R: resveratrol; and CFU: colony-forming units; numbers refer to the weight percentage of extract used in each film.

**Table 2:** Results of antimicrobial tests.

Code Sample	Aerobic Mesophilic Bacteria		Coliforms		Fungi	
	Approved Test	Inhibition Zone, mm	Approved Test	Inhibition Zone, mm	Approved Test	Inhibition Zone, mm
QS2	0/6	0 ± 0	1/6	2 ± 0.89	2/6	1.333 ± 0.52
QSA0.5	5/6	2.333 ± 0.52	4/6	2.333 ± 0.52	3/6	1.333 ± 0.52
QSA2	5/6	2.5 ± 0.55	4/6	2.5 ± 0.55	3/6	3.5 ± 1.22
QSA5	4/6	2.5 ± 0.55	5/6	2.5 ± 0.55	5/6	8 ± 2.76
QSAm0.5	2/6	3.667 ± 0.52	4/6	1.5 ± 0.55	1/6	6.167 ± 2.40
QSAm2	4/6	9.333 ± 1.03	3/6	3.333 ± 0.52	4/6	6.5 ± 2.35
QSAm5	4/6	9.5 ± 0.84	4/6	3.5 ± 0.55	4/6	7 ± 2.76
QSB0.5	4/6	1.167 ± 0.41	1/6	2.667 ± 0.82	4/6	7 ± 2.76
QSB2	4/6	1.333 ± 0.52	1/6	2.833 ± 0.98	5/6	7.333 ± 2.58
QSB5	4/6	2.5 ± 0.55	1/6	3 ± 0.89	5/6	7.5 ± 2.59
QSG0.5	2/6	2.333 ± 0.82	1/6	1.833 ± 0.75	2/6	7.333 ± 2.58
QSG2	5/6	4 ± 0.89	3/6	2.167 ± 0.75	0/6	7.333 ± 2.58
QSG5	6/6	4.167 ± 0.98	4/6	2.167 ± 0.75	1/6	7.667 ± 2.58
QSO0.5	3/6	4 ± 0.89	2/6	1.5 ± 0.55	2/6	1.5 ± 0.55
QSO2	5/6	4.167 ± 0.75	4/6	1.5 ± 0.55	3/6	1.667 ± 0.52
QSO5	6/6	4.167 ± 0.98	4/6	2 ± 0.63	4/6	1.667 ± 0.52
QSP0.5	5/6	4 ± 0.89	1/6	1.833 ± 0.41	4/6	1.833 ± 0.41
QSP2	3/6	3.5 ± 0.84	1/6	1.333 ± 0.52	3/6	1.333 ± 0.52
QSP5	1/6	3.333 ± 0.82	2/6	1.333 ± 0.52	0/6	1.333 ± 0.52
QSR0.5	2/6	1.833 ± 0.98	5/6	6.5 ± 2.35	3/6	2.5 ± 0.84
QSR2	3/6	1.833 ± 0.75	2/6	7.167 ± 2.79	3/6	6.833 ± 2.93
QSR5	4/6	2 ± 0.89	4/6	8 ± 2.76	3/6	7.167 ± 2.78

“Approved test” means that the films could inhibit microorganism growth in its periphery during the test period. The inhibition zone indicated for each film is related to the positive results shown during antimicrobial activity.

**Table 3:** Average film thickness.

Sample	Average Thickness, mm	Sample	Average Thickness, mm
QS2	0.164 ± 0.013	QSG2	0.226 ± 0.009
QSA0.5	0.194 ± 0.011	QSG5	0.254 ± 0.025
QSA2	0.214 ± 0.019	QSO0.5	0.190 ± 0.01
QSA5	0.238 ± 0.013	QSO2	0.202 ± 0.013
QSAm0.5	0.212 ± 0.015	QSO5	0.194 ± 0.005
QSAm2	0.244 ± 0.017	QSP0.5	0.266 ± 0.021
QSAm5	0.248 ± 0.043	QSP2	0.216 ± 0.011
QSB0.5	0.154 ± 0.005	QSP5	0.272 ± 0.033
QSB2	0.162 ± 0.018	QSR0.5	0.184 ± 0.011
QSB5	0.226 ± 0.015	QSR2	0.174 ± 0.005
QSG0.5	0.236 ± 0.011	QSR5	0.238 ± 0.011

Precision = 0.01 mm.

**Table 4:** Transmittance percentages (%*T*) at 280 nm and 400 nm, transparency, and opacity of each film.

Sample	%T at 280 nm	% T at 400 nm	Transparency	Opacity
QS2	10.2442 ± 0.71	30.8092 ± 2.34	9.74382 ± 0.19	0.07681 ± 0.01
QSA0.5	0.0866 ± 0.070	3.94262 ± 1.78	7.03310 ± 0.52	0.12132 ± 0.0017
QSA2	0.9668 ± 0.39	11.7966 ± 1.54	6.61022 ± 0.19	0.11328 ± 0.0084
QSA5	0.5295 ± 0.12	16.8562 ± 1.90	6.50092 ± 0.20	0.11983 ± 0.0074
QSAm0.5	1.0697 ± 0.33	16.5411 ± 1.85	6.98136 ± 0.21	0.13173 ± 0.02
QSAm2	0.2182 ± 0.18	8.8739 ± 1.33	5.59052 ± 0.09	0.20691 ± 0.01
QSAm5	0.3032 ± 0.15	7.9730 ± 0.82	5.39832 ± 0.26	0.21030 ± 0.02
QSB0.5	2.0982 ± 0.78	25.4562 ± 3.24	10.3050 ± 0.27	0.07983 ± 0.02
QSB2	0.2811 ± 0.13	11.4734 ± 2.14	9.34763 ± 0.24	0.11238 ± 0.01
QSB5	0.0566 ± 0.05	1.1172 ± 0.22	4.80696 ± 0.29	0.33784 ± 0.02
QSG0.5	0.4638 ± 0.15	12.8224 ± 0.54	6.37701 ± 0.07	0.13510 ± 0.01
QSG2	0.9107 ± 0.22	17.0909 ± 1.57	6.8277 ± 0.051	0.13645 ± 0.01
QSG5	0.1769 ± 0.23	1.6192 ± 0.82	5.18060 ± 0.67	0.25293 ± 0.01
QSO0.5	2.0135 ± 0.47	17.5357 ± 2.39	7.83758 ± 0.25	0.08531 ± 0.0039
QSO2	0.4690 ± 0.18	19.3792 ± 1.36	8.50917 ± 0.13	0.07273 ± 0.0058
QSO5	0.2167 ± 0.29	5.6792 ± 1.50	8.21114 ± 0.08	0.10168 ± 0.0064
QSP0.5	0.2437 ± 0.40	3.2979 ± 0.40	5.9859 ± 0.18	0.17956 ± 0.02
QSP2	0.0272 ± 0.029	0.1323 ± 0.10	6.7034 ± 0.52	0.20245 ± 0.02
QSP5	0.1623 ± 0.10	0.1195 ± 0.12	4.0516 ± 0.38	0.55747 ± 0.07
QSR0.5	8.7779 ± 2.05	27.2905 ± 3.38	8.4157 ± 0.25	0.09657 ± 0.0077
QSR2	10.9962 ± 4.31	26.4296 ± 9.05	6.6499 ± 0.81	0.09698 ± 0.02
QSR5	4.7594 ± 0.22	31.5367 ± 3.17	7.0720 ± 0.16	0.09806 ± 0.01

**Table 5:** pH values of each reagent necessary for the film synthesis.

Compound	pH
Acetic acid at 1% (*v*/*v*)	2.60 ± 0.028
Chitosan at 2% (*w*/*v*)	4.46 ± 0
Starch at 2% (*w*/*v*)	5.82 ± 0.014
Cranberry extract	2.86 ± 0.028
Blueberry extract	3.29 ± 0.014
Beetroot extract	4.31 ± 0.014
Pomegranate extract	3.11 ± 0.014
Oregano extract	5.24 ± 0
Pitaya extract	5.14 ± 0.014
Resveratrol extract	4.93 ± 0.014

**Table 6:** pH values of chitosan–starch films at day 1 and day 15.

Sample	Day 1	Day 15
	pH Meter	pH Meter
QS2	4.57 ± 0.007	5.29 ± 0.021
QSA0.5	4.73 ± 0.007	5.86 ± 0.014
QSA2	4.70 ± 0.007	5.54 ± 0.007
QSA5	4.68 ± 0.014	5.33 ± 0.028
QSAm0.5	4.11 ± 0.007	4.58 ± 0.014
QSAm2	4.07 ± 0.007	4.47 ± 0.028
QSAm5	4.02 ± 0.007	4.34 ± 0.007
QSB0.5	4.83 ± 0.007	5.88 ± 0.007
QSB2	4.78 ± 0.007	5.60 ± 0.028
QSB5	4.62 ± 0.014	5.59 ± 0.007
QSG0.5	4.52 ± 0.021	6.14 ± 0.014
QSG2	4.41 ± 0	6.02 ± 0.035
QSG5	4.31 ± 0.014	5.97 ± 0.007
QSO0.5	4.81 ± 0.007	5.89 ± 0.014
QSO2	4.77 ± 0.014	5.58 ± 0.035
QSO5	4.62 ± 0.014	5.52 ± 0.007
QSP0.5	4.01 ± 0.007	5.26 ± 0.007
QSP2	4.04 ± 0.014	5.46 ± 0.014
QSP5	4.09 ± 0.014	4.83 ± 0.014
QSR0.5	4.65 ± 0.007	5.92 ± 0.021
QSR2	4.62 ± 0.007	5.88 ± 0.007
QSR5	4.59 ± 0.007	5.77 ± 0.028

**Table 7:** Multi-factor ANOVA interpretation.

Factor	*p* Value	Statistical Implication	Conclusion
AOX	<0.0005	Reject null hypothesis of no difference in means.	At least one mean pH is significantly different from the rest because of the type of antioxidant that was incorporated into the film.
% AOX	0.002	Reject null hypothesis of no difference in means.	At least one mean pH is significantly different from the rest because of the content of antioxidant (% *wt*) in the film.
Day	<0.0005	Reject null hypothesis of no difference in means.	The mean pH measured on day 1 is significantly different from that measured on day 15.
AOX and % AOX	0.991	Do not reject null hypothesis of no interaction between the factors.	The effect of the type of antioxidant on the mean pH does not significantly depend on its content (% *wt*).
AOX and day	<0.0005	Reject null hypothesis of no interaction between the factors.	The effect of the type of antioxidant on the mean pH significantly depends on the day when the pH is measured.
% AOX and day	0.175	Do not reject null hypothesis of no interaction between the factors.	The effect of the content of antioxidant (% *wt*) on the mean pH does not significantly depend on the day when the pH is measured.
AOX, % AOX and day	<0.0005	Reject null hypothesis of no interaction among the factors.	There is a significant effect of the combination of the three factors in the mean pH values.

AOX: antioxidant; *% wt*: weight percentage.

**Table 8:** Storage modulus (E’), tan delta (tan δ), and glass transition temperature (*T*g) of the films.

Sample	Storage Modulus (E’)		*T*g °C	Tan δ
	Average Value (MPa)		Average Value	
	50 °C	100 °C		
QS2	4753	3093	185.2	0.5405
QSA0.5	3001	1967	158.4	0.330
QSA2	3868	2633	90.1	0.166
QSA5	2982	2065	87.1	0.151
QSAm5	2214	497.17	104	0.361
QSG5	2407	665	102	0.331
QSO5	494	250	93.3	0.459
QSP0.5	1668	838	102.1	0.321
QSP5	925	376	95.3	0.364
QSR5	441	179	88.6	0.542
